# Identification, molecular characterization and expression of *JAZ* genes in *Lycoris aurea*

**DOI:** 10.1371/journal.pone.0230177

**Published:** 2020-03-17

**Authors:** Peng Wang, Shuojun Yu, Xiaokang Han, Junya Xu, Qingyuan He, Sheng Xu, Ren Wang

**Affiliations:** 1 Institute of Botany, Jiangsu Province and Chinese Academy of Sciences, Nanjing, China; 2 College of Life and Health Science, Anhui Science and Technology University, Fengyang, China; 3 State Key Laboratory of Genetic Engineering, School of Life Sciences, Fudan University, Shanghai, China; 4 The Jiangsu Provincial Platform for Conservation and Utilization of Agricultural Germplasm, Nanjing, China; CSIR-Central Institute of Medicinal and Aromatic Plants, INDIA

## Abstract

Jasmonates (JAs) are key phytohormones involved in regulation of plant growth and development, stress responses, and secondary metabolism. It has been reported that treatments with JAs could increase the contents of Amaryllidaceae alkaloids in Amaryllidaceae plants. Jasmonate ZIM (zinc-finger inflorescence meristem) domain (JAZ) proteins are key components in JA signal processes. However, JAZ proteins have not been characterized in genus *Lycoris*. In this study, we identified and cloned seven differentially expressed *JAZ* genes (namely *LaJAZ1*–*LaJAZ7*) from *Lycoris aurea*. Bioinformatic analyses revealed that these seven LaJAZ proteins contain the ZIM domain and JA-associated (Jas, also named CCT_2) motif. Quantitative reverse transcription polymerase chain reaction (qRT-PCR) analysis revealed that these *LaJAZ* genes display different expression patterns in *L*. *aurea* tissues, and most of them are inducible when treated with methyl jasmonate (MeJA) treatment. Subcellular localization assay demonstrated that LaJAZ proteins are localized in the cell nucleus or cytoplasm. In addition, LaJAZ proteins could interact with each other to form homodimer and/or heterodimer. The findings in this study may facilitate further functional research of the *LaJAZ* genes, especially the potential regulatory mechanism of plant secondary metabolites including Amaryllidaceae alkaloids in *L*. *aurea*.

## Introduction

Jasmonates (JAs), including jasmonic acid (JA) and related precursors and derivatives, are important phytohormones that regulate plant growth and development, metabolism and responses to biotic and abiotic stresses [1–3]. Vascular plants generate JA via the octadecanoid and hexadecanoid biosynthetic pathway from polyunsaturated fatty acids to finally accumulate the bioactive jasmonoyl-L-isoleucine (JA-Ile) molecule, the (+)-7-*iso*-JA-Ile [4–6]. JA-Ile triggers an interaction between the F-box CORONATINE INSENSITIVE 1 (COI1) and the jasmonate-ZIM (zinc-finger inflorescence meristem) domain (JAZ) family proteins, leading to the ubiquitination and subsequent degradation of the JAZ proteins via 26S proteasome [7]. Because of lacking key enzymes of the JA-Ile biosynthetic pathway, bryophytes such as *Marchantia polymorpha* are unable to synthesize JA-Ile, of which dinor 12-oxo-phytodienoic acid (dn-OPDA) acts as the COI1-JAZ ligand [7,8]. Since the JAZ proteins function as repressors of several transcription factors (TFs) for jasmonate-inducible genes, upon perception of a jasmonate signal, the ubiquitination and degradation of JAZ repressors relief TFs that in turn allow the cellular transcription reprogramming [1–4, 9,10].

Acting as both repressors of TFs and co-receptors of JA-Ile, JAZ proteins are present in all land plants, from bryophytes to eudicots [7,11,12]. For instance, 13 JAZ proteins have been identified in *Arabidopsis* [13,14]. There are 15 members of JAZ proteins in rice [15]. More recently, the single *JAZ* gene (*MpJAZ*) in the liverwort *Marchantia polymorpha* was functionally characterized [12]. The JAZ proteins belong to plant-specific TIFY family, which also contains TIFY, PEAPOD (PPD) and ZIM-like (ZML) protein subfamilies [11,13]. JAZ proteins contain a conserved ZIM domain (TIF[F/Y]XG) near the N-terminal region [4,10,11,13], and a JA-associated (Jas, also named CCT_2) motif at the C-terminal region [11,16]. In the absence of JA, JAZ proteins recruit the general co-repressors TOPLESS (TPL) complex as well as TPL-related proteins (TPRs) via the specific adaptor protein Novel Interactor of JAZ (NINJA) to repress the activity of TFs [17–19]. Besides, a minority of non-canonical JAZ proteins (such as *Arabidopsis* JAZ7, JAZ8 and JAZ13) have been reported to contain ethylene-responsive element binding factor (ERF)-associated amphiphilic repression (EAR) domains and recruit TPL/TPRs independently of NINJA [14,20,21].

*Lycoris aurea* (L’ Her.) Herb, is an ornamentally and medicinally important perennial herbaceous plant, belonging to the Amaryllidaceae family, from which there were a variety of Amaryllidaceae alkaloids found to exhibit medicinal values [22]. Previous studies of *L*. *aurea* have mainly focused on karyotype [23–25], physiological analysis [26,27], chemical composition [28–30], medicinal usage [31,32], and molecular aspects for gene cloning [33–36]. For example, several enzymes involved in Amaryllidaceae alkaloids biosynthesis, including cinnamate 4-hydroxylase [34], tyrosine decarboxylase [35] and norbelladine 4’-*O*-methyltransferase [36] have been identified. Besides, although the induction effects of exogenous methyl jasmonate (MeJA) treatments on Amaryllidaceae alkaloids accumulation has been reported [37,38], little is known about the possible transcriptional or post-transcriptional regulation mechanism of this biological process in *L*. *aurea*. Recently, the transcriptome and small RNA sequencing under MeJA-treated *L*. *aurea* have been performed [39,40], which provided us a basic database to identify the key genes potentially involved in regulating the biological processes (especially Amaryllidaceae alkaloids biosynthesis) of *L*. *aurea*. Hence, in this study, based on our previous transcriptome data of *L*. *aurea* treated with MeJA [39], seven *JAZ* genes (*LaJAZ1*-*LaJAZ7*) were isolated and cloned. Also, the expression profiles of these genes in different tissues and their response to MeJA were analyzed. In addition, our data demonstrated that the identified LaJAZ proteins were localized in nucleus or cytoplasm, and formed heterodimers and homodimers as well. Our results may provide a basis to elucidate the JA signalling pathway in *Lycoris* species.

## Materials and methods

### Plant growth conditions and treatments

The seeds of *L*. *aurea* were surface sterilized and germinated in petri dishes with half-strength Murashige and Skoog (MS) medium (pH 5.8) at 25 °C in the dark for 10 days, and then cultured in a growth chamber at 25 °C under a 14/10 h day/night rhythm. For *LaJAZ* gene expression analysis, different tissues of *L*. *aurea* including root, bulb, and leaf were taken at vigorous vegetative growth stage, but flower stalk and flower from the same sampling plants were collected during the flowering time. For MeJA treatment, one-year-old seedlings were imposed in 0.1 mM MeJA for 0, 6, 12, 24, and 36 h. Seedlings grown in MeJA-free solution (dissolved 1% DMSO) were used as control. The seedlings were harvested from at least three representative plants, immediately frozen in liquid nitrogen and then stored at −80 °C until further use.

### RNA isolation and cDNA synthesis

Total RNA was isolated with the RNAprep Pure Plant Kit (Tiangen Biotech, Beijing, China) from 200 mg of *L*. *aurea* samples, and purified with RNase-free DNase I according to the manufacturer's instructions. First strand cDNA synthesis was performed on 2 μg RNA using PrimeScript^™^ 1st strand cDNA Synthesis Kit (TaKaRa, Dalian, China) with oligo (dT) _18_ and random hexamer primers.

### Isolation of *L*. *aurea* LaJAZ family genes

In our previous transcriptomics study, two sequencing cDNA libraries of *L*. *aurea* prepared from MeJA-free (control) and MeJA-treated samples (for 6 h) were sequenced [39]. Here, by using BioEdit software (http://www.mbio.ncsu.edu/BioEdit/bioedit.html), a local library for *L*. *aurea* non-redundant unigenes assembled from the two cDNA libraries was created. Each JAZ protein sequence of *Arabidopsis* and rice was locally Blasted against the library using the tblastn program in BioEdit. Sequences with higher coverage and identity, and with lower e-value were selected for *L*. *aurea JAZ* gene cloning (S1 Table). According to these sequences, a primer set was designed (S2 Table). The full-length cDNA of *LaJAZ* gene was amplified and sequenced.

### Sequence analysis and phylogenetic tree construction

The sequences of the *LaJAZ* genes were translated and analyzed by open reading frame (ORF) Finder (https://www.ncbi.nlm.nih.gov/orffinder/). Their isoelectric points (pIs) were predicted by using ExPASy (http://us.expasy.org/tools). The conserved domains of presumed proteins were predicted by using Conserved Domain Database of NCBI (http://www.ncbi.nlm.nih.gov/Structure/cdd/wrpsb.cgi), MOTIF search (http://www.genome.jp/tools/motif/) and the SMART program (http://smart.embl-heidelberg.de/). TIFY domain and Jas motif were identified through multiple sequence alignment by Clustal Omega [41] and visualized by Jalview software. For phylogenetic analysis, the amino acid sequences of JAZ proteins involved in different plant species (S3 Table) were constructed by using MEGA software (version 5.0) using the maximum likelihood method (1000 bootstrap replicates). Phylogenetic trees were visualized by iTOL online tool (https://itol.embl.de/).

### Subcellular localization analysis

The complete ORF of *LaJAZ* genes without the termination codon was amplified by PCR using specific primers (S2 Table). The PCR products were then assembled to the linear expression vector pAN580 for N-terminal green fluorescent protein (GFP) fusion by ClonExpress One Step Cloning Kit (Vazyme Biotech, Beijing, China). For the construction of a nucleus-localized marker, the coding sequence of *HMGB1* gene (At3g51880) was amplified using specific primers (S2 Table). After digesting with *Bam*HI and *Sma*I, the PCR product was inserted into the modified vector P16ΔS:sXVE:mCherry for N-terminal mCherry fusion. The transient expression of GFP and mCherry fusion proteins in *Arabidopsis* mesophyll protoplasts was performed following the method described previously [42], and then observed under a laser scanning confocal microscope (LSM710 META, Carl Zeiss, Germany).

### Western blot

Proteins were extracted by homogenizing transfected protoplast cells in 200 μL of lysis buffer (50 mM Tris-HCl, 150 mM NaCl, 1 mM EDTA, 1 mM PMSF, 10% glycerol, 25 mM β-glycerophosphate, pH 7.5). The homogenates were centrifuged at 16,000 × g for 20 min at 4 °C, then the supernatants were collected and proteins were separated by SDS-polyacrylamide gel electrophoresis (SDS-PAGE) and then electro-transferred onto polyvinvlidene difluoride (PVDF) membranes (Millipore, Billerica, MA, USA). Membranes were blocked in a 3% BSA/TBST buffer solution for 2 h at room temperature, followed by incubation with anti-GFP rabbit polyclonal antibody (Sangon Biotech, Shanghai, China) at 4 °C overnight. Following incubation, the membranes were washed three times with TBST buffer and incubated with a horseradish peroxidase (HRP)-conjugated mouse anti-rabbit secondary antibody (Sangon Biotech, Shanghai, China). The blots were washed again three times with TBST buffer and the immunoreactive bands were visualized using the standard HRP/3,3’-diaminobenzidine (DAB) method.

### Quantitative reverse transcription PCR (qRT-PCR) analysis

In order to determine the expression levels of *LaJAZ* genes in different tissues and under MeJA treatments, total RNAs were extracted as described above, and qRT-PCR was performed. Gene-specific primers for *LaJAZ* genes were designed and synthesized (S2 Table). The relative expression values were normalized by using *L*. *aurea* IP41-like protein gene (*TIP41*) as the reference, and transformed to a log2 scale [43].

### Yeast two-hybrid (Y2H) assay

The Y2H assay was performed using the Matchmaker^™^ Gold Yeast Two-Hybrid system (Clontech, Mountain View, CA, USA). The coding sequences of *LaJAZ* genes were amplified by PCR with gene-specific primers (S2 Table), and cloned into the yeast two-hybrid vectors pGBKT7 (bait vector) or pGADT7 (prey vector). The bait vector and prey vector were transformed into Y2HGold and Y187 yeast strains, respectively. The interactions between these proteins after mating were determined by the growth on DDO medium (SD/–Trp/–Leu/) and QDO medium (SD/–Trp/–Leu/–His/–Ade) with 5-bromo-4-chloro-3-indoxyl α-_D_-galactoside (X-α-Gal) assay according to the instruction manual.

### Statistical analysis

Values are means ± standard error (SE) of three independent experiments with at least three replicates for each. Differences among treatments were analyzed by one-way ANOVA, taking *P* < 0.05 as significant according to Duncan’s multiple range test.

## Results

### Identification and molecular cloning of *JAZ* genes in *L*. *aurea*

In order to identify ortholog *JAZ* genes in *L*. *aurea*, a tblastn search against our previous transcriptome database [39] was performed using *Arabidopsis* and rice JAZs protein sequences as query templates. At least 9 *TIFY* genes containing different unigenes (or contigs) were searched in *L*. *aurea* transcriptome database (S1 Table). After PCR amplification and sequencing validation, seven full-length cDNAs of the *LaJAZ* genes containing both the ZIM domain (TIFY motif) and Jas motif (also named as CCT_2 motif) were verified. In addition, full-length cDNA of LaTIFY1 and LaTIFY2 was also characterized (S1 Table).

### Sequence and phylogenetic analysis of LaJAZ proteins

**Fig 1:**
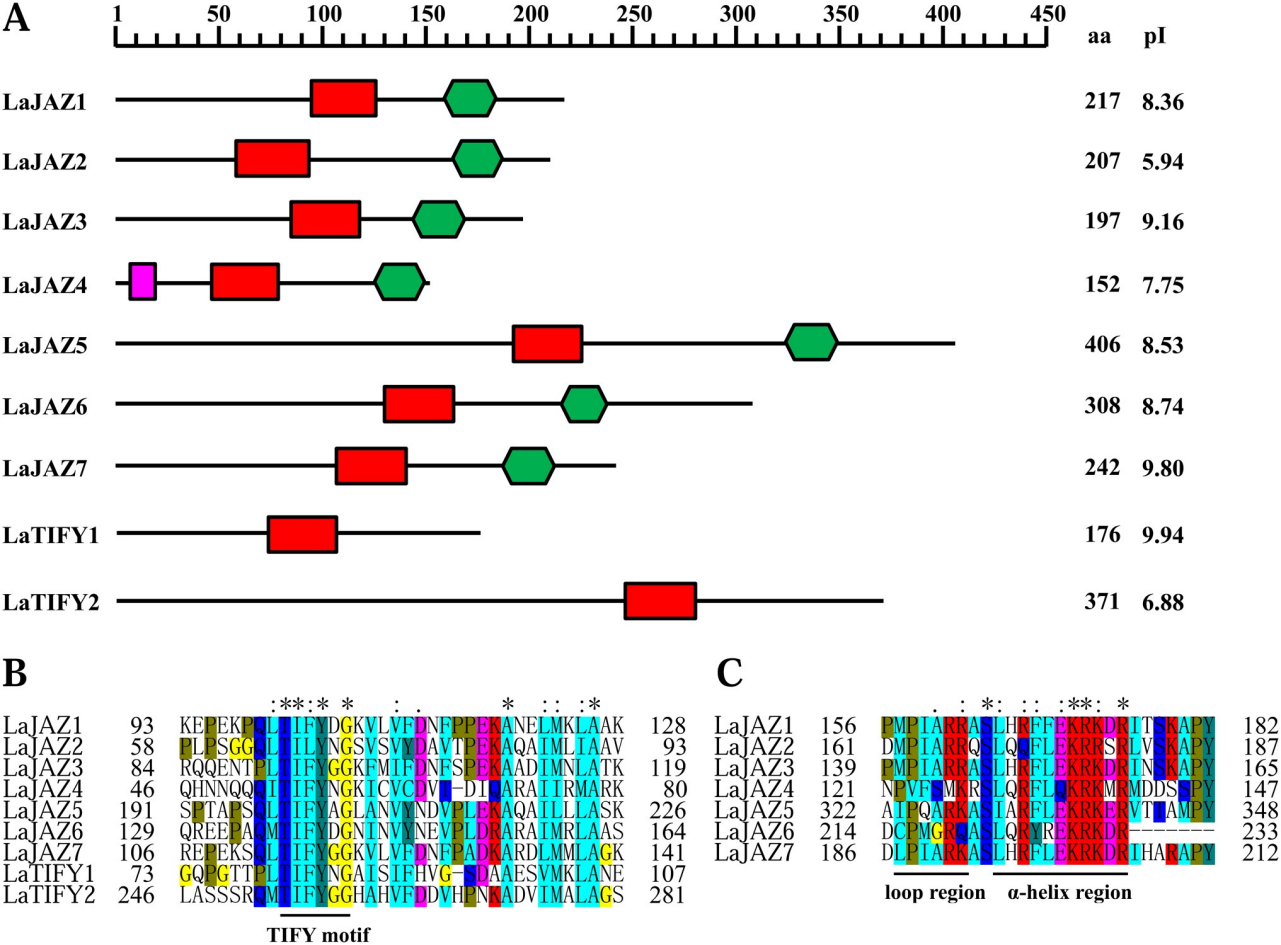
The domain structure of the corresponding LaJAZ and LaTIFY proteins. (A) The conserved protein motifs were presented among the LaTIFY1, LaTIFY2 and seven LaJAZ proteins. Purple box: EAR domain; Red box: TIFY domain; Green box: Jas motif. aa: the number of amino acids; pI: isoelectric point. Sequence alignment of the conserved ZIM domain (B) and Jas motif (C) of *L*. *aurea* TIFY1, TIFY2 and JAZ proteins was made by Clustal Omega software.

**Fig 2:**
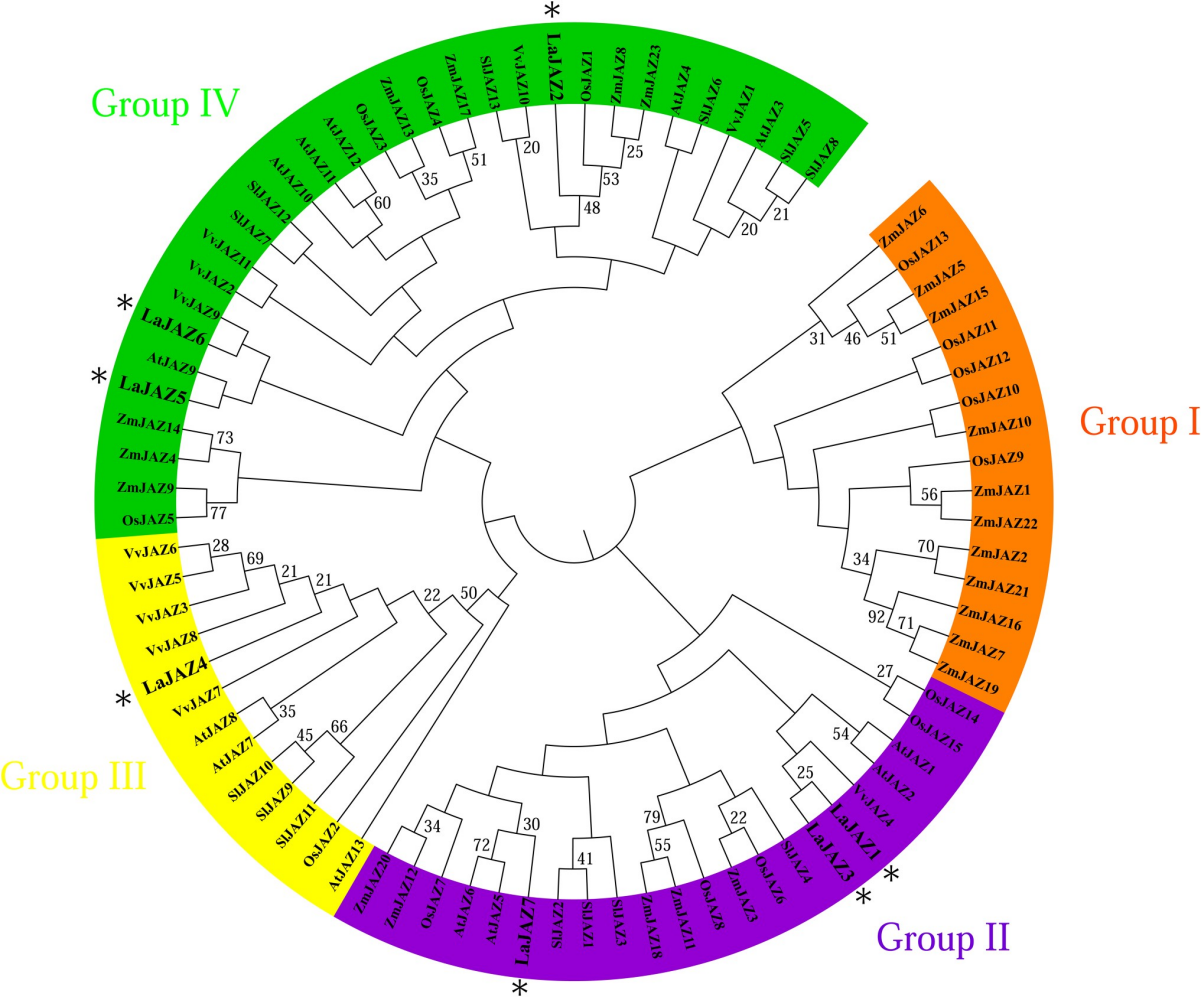
Phylogenetic relationships between LaJAZ proteins, and the JAZ proteins from *Arabidopsis thaliana*, *Zea mays*, *Oryza sativa*, *Solanum lycopersicum* and *Vitis vinifera*. The maximum-likelihood method with 1000 bootstrap replications was used. GenBank accession numbers were listed in S3 Table.

As shown in Fig 1A, the protein length of LaJAZ1–LaJAZ7 varies in a range from 152 to 406 amino acids. LaJAZ5 is the longest among these seven LaJAZ proteins. Also, the pI features of most LaJAZ proteins were >7 (except for LaJAZ2 with pI of 5.94), indicating that most of LaJAZ proteins should be basic proteins (Fig 1A). Although multiple alignment analysis showed that the seven LaJAZ proteins shared only 21.81% identity at the amino acid sequence level, two identified sequence motifs (TIFY and Jas) are highly conserved in all seven LaJAZ proteins (Fig 1B and 1C). In addition, an EAR motif was observed only in LaJAZ4 (S1 Fig). Further, based on sequence alignments of LaJAZ proteins and other plant JAZ proteins, a phylogenetic tree was created (Fig 2) to show that the JAZ proteins were clustered into four branches (Groups I–IV). The seven LaJAZs were distributed into three branches, such as group II including LaJAZ1, LaJAZ3 and LaJAZ7, group III containing LaJAZ4 and group IV having LaJAZ2, LaJAZ5 and LaJAZ6.

### Tissue expression patterns of the *LaJAZ* genes

**Fig 3:**
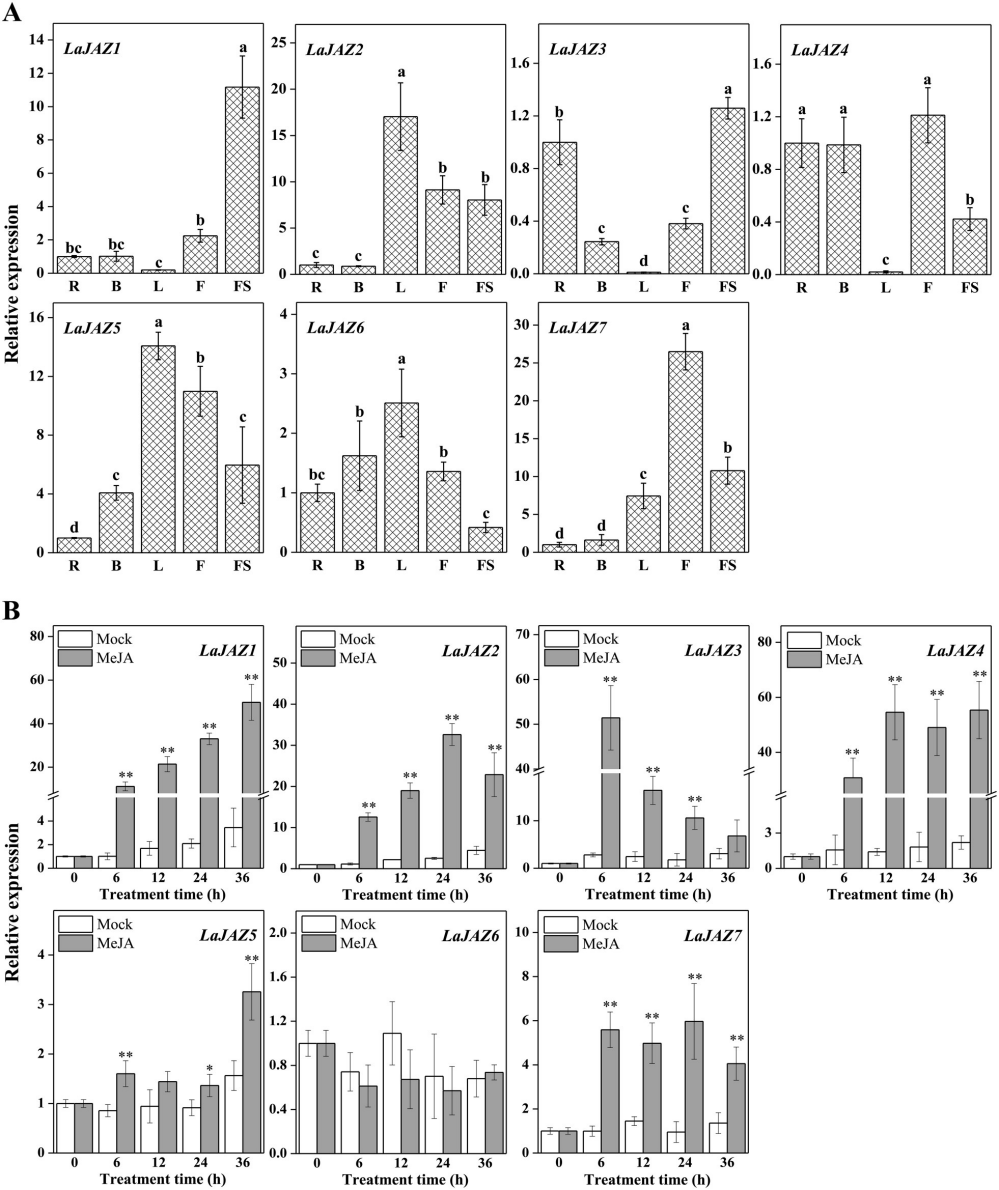
Expression analysis of seven *LaJAZ* genes in different tissues (A) and under MeJA treatments (B) by qRT-PCR. Different lowercase letters indicate a significant difference, as determined by the Duncan’s multiple range test (*p*-value < 0.05). Asterisk indicates a significant difference between control versus treatment according to student's t-test. R: Root; L: Leaf; B: Bulb; F: Flower; FS: Flower stalk.

For detecting *LaJAZ* transcript signals to profile the gene expression pattern in *L*. *aurea*, we carried out qRT-PCR assays to measure the relative amount of corresponding transcripts of *LaJAZ* genes in different plant tissues. As shown in Fig 3A, in general, seven *LaJAZ* genes expressed in all the five detected tissues (root, bulb, leaf, flower stalk, and flower) of *L*. *aurea*, and most *LaJAZ* genes show higher expression in flower than that in bulb (Fig 3A). Moreover, low expression levels of *LaJAZ1* and *LaJAZ3* were detected in leaf, whereas the high expression levels of both genes were in flower stalk. *LaJAZ4* also showed its expression at the lowest level in leaf, but accumulated relatively high levels in root, bulb and flower. In contrast, *LaJAZ2*, *LaJAZ5* and *LaJAZ6* were all highly expressed in leaf, but exhibited different low expression patterns. For example, *LaJAZ2* exhibited low expression levels in both root and bulb, while *LaJAZ5* showed its lowest expression levels in root. In addition, *LaJAZ6* was expressed at low level in flower stalk. The highest expression level of *LaJAZ7* was observed in flower, whereas the lowest transcript was in root (Fig 3A).

### Expression of *LaJAZ* genes in response to MeJA treatment

Following the profiling of the gene expressions in *L*. *aurea* tissues, we also wondered whether the expression of these seven *LaJAZ* genes were responsive to the jasmonate treatment in *L*. *aurea* seedling. As shown in Fig 3B, in general, the transcription of 6 *LaJAZ* genes, except *LaJAZ6*, would be induced in the seedlings treated with MeJA, while their expression patterns much varied. For example, the expression of *LaJAZ1*, *LaJAZ4* and *LaJAZ5* increased from 6 h and reached to the highest level at 36 h after MeJA elicitation. Transcripts of *LaJAZ3* and *LaJAZ2* increased to their highest level at 6 h and 24 h, respectively after MeJA treatment. *LaJAZ7* was also up-regulated after MeJA treatment, while its expression would delay since a peak of its transcript accumulation occurred at 24 h. Our results indicated that the expression pattern of most *LaJAZ* genes was in good agreement with our previous transcriptome data [39], except *LaJAZ2* (S1 Table).

### Subcellular localization of LaJAZ proteins

**Fig 4:**
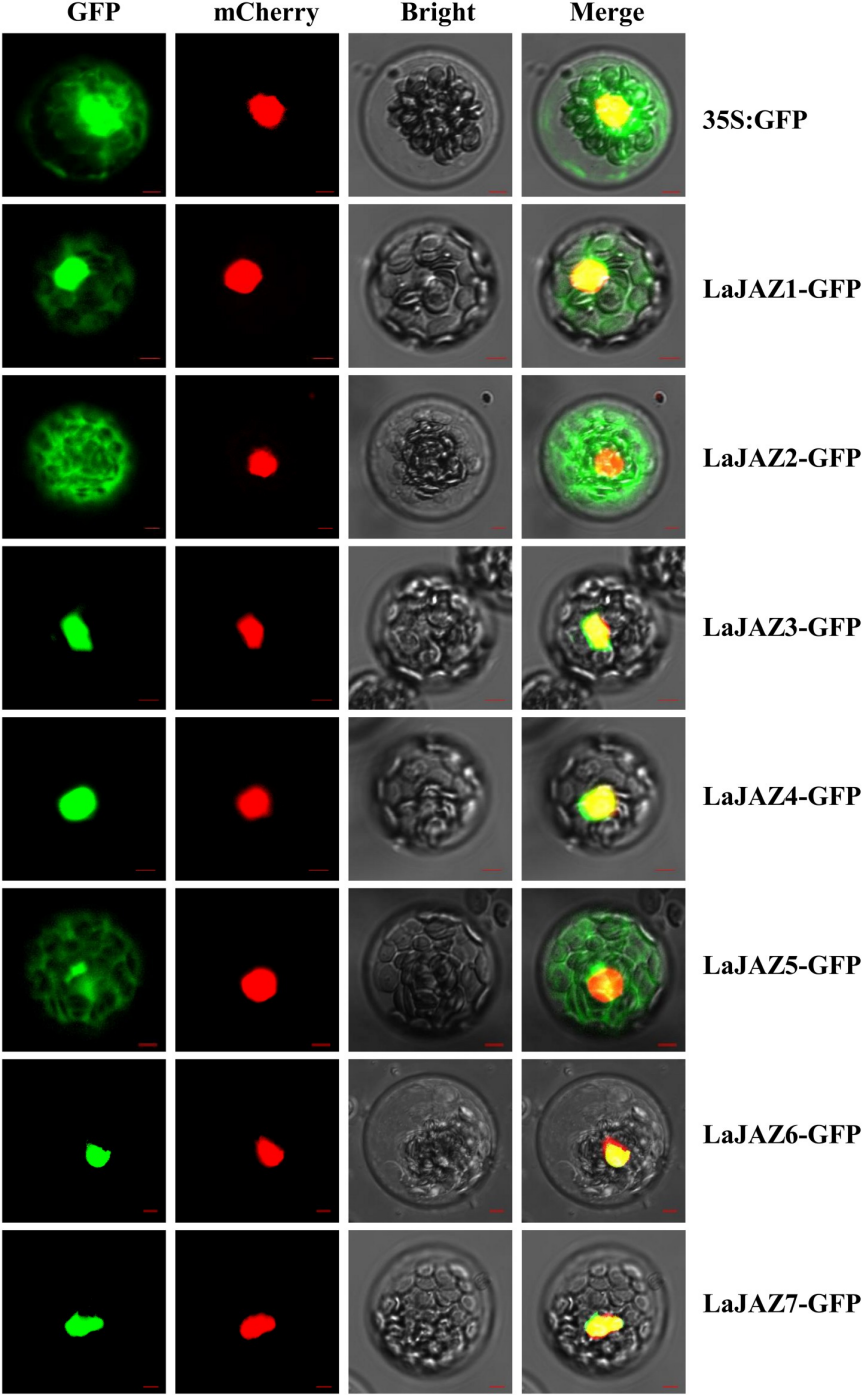
Subcellular localization analysis of LaJAZ-GFP fusion protein transiently expressed in *Arabidopsis* protoplasts. The photographs were taken of green fluorescence (GFP), red fluorescence (chlorophyll), visible light and merged light. Bar = 5 μm.

To determine the subcellular localization of the LaJAZ proteins in plant cell, The ORFs of *LaJAZ1*–*LaJAZ7* were fused with GFP under the control of CaMV 35S promoter, respectively (S2 Fig). *Arabidopsis* HMGB1 in fusion with mCherry protein (HMGB1-mCherry) was used for the nucleus-localized marker [44]. After each LaJAZ-GFP construct combined with HMGB1-mCherry were introduced in *Arabidopsis* protoplasts, the LaJAZ-GFP and HMGB1-mCherry signals were viewed individually. The subcellular localization results showed that LaJAZ3-GFP, LaJAZ4-GFP, LaJAZ6-GFP and LaJAZ7-GFP were identical to that of HMGB1-mCherry, clearly showing that all of them were localized to the nucleus. Meanwhile, the fluorescence of LaJAZ1-GFP was observed in both nucleus and cytoplasm, which was similar to the observation with non-targeted GFP in the protoplasts. In addition, LaJAZ2-GFP and LaJAZ5-GFP was observed in a cytosolic fluorescence pattern (Fig 4). On the other hand, transfected protoplasts were also analyzed for the expression of LaJAZ1-GFP, LaJAZ2-GFP and LaJAZ5-GFP by western blot with an anti-GFP polyclonal antibody to assess if the protein size corresponds to the JAZ fused to GFP. As expected, the band size of LaJAZ1-GFP and LaJAZ2-GFP was approximately corresponding to the molecular weight of the GFP protein plus that of LaJAZ1 and LaJAZ2, respectively (S3 Fig). Unfortunately, the band of LaJAZ5-GFP was not detectable, which might be due to the lower transfection efficiency of LaJAZ5-GFP in protoplast.

### Homo- and heterodimeric interaction of LaJAZ proteins

**Fig 5:**
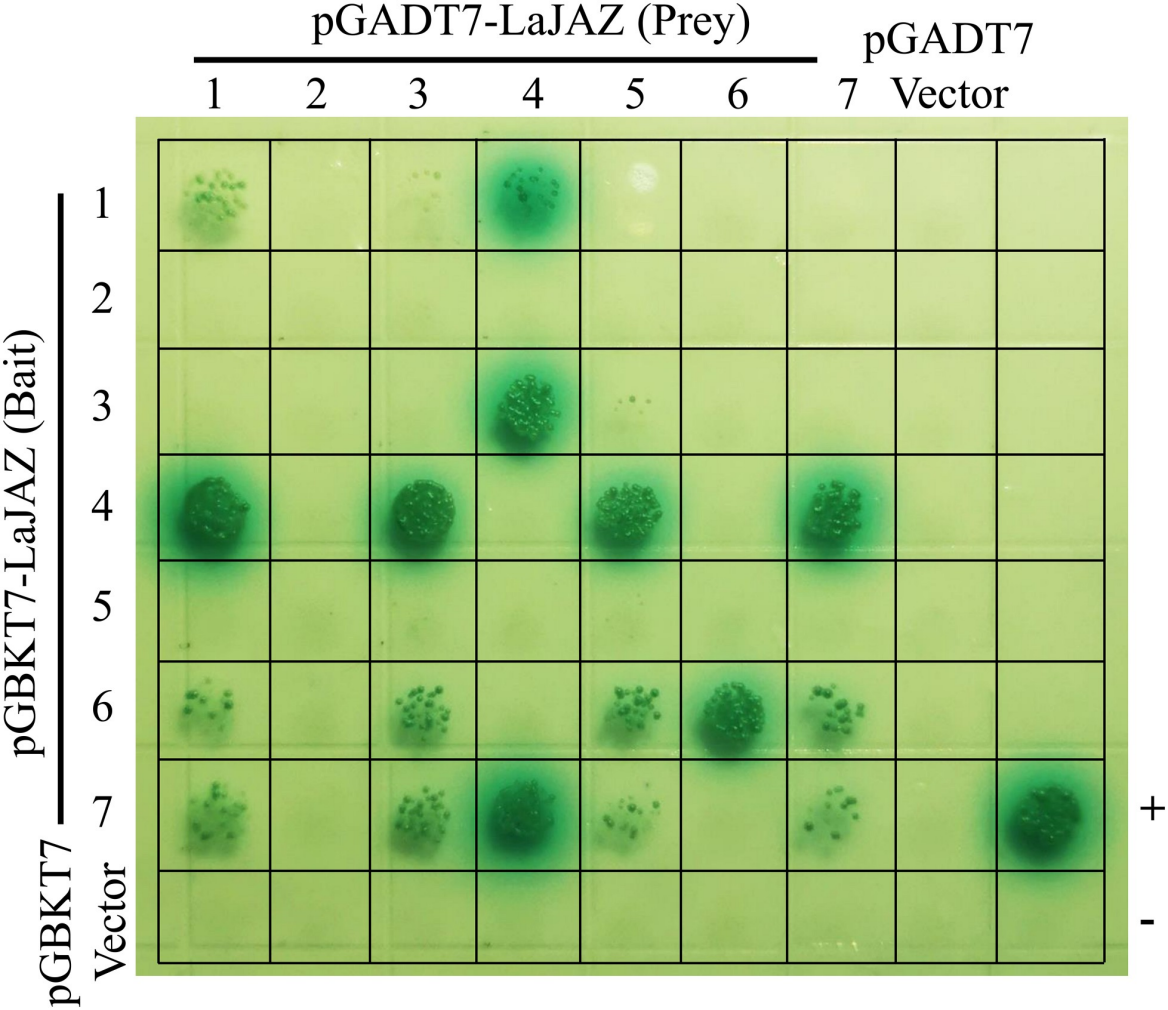
Yeast two-hybrid assay of interactions among LaJAZ proteins. Interactions among the LaJAZ proteins were analyzed by yeast mating for 3 days. Positive transformants were determined on QDO (SD-Trp/-Leu/-Ade/-His) nutritional selection medium supplemented with 5-bromo-4-chloro-3-indoxyl α-d-galactoside (X-α-Gal). The mating of pGBKT7-53 in Y2H gold yeast plus pGADT7-SV40 in Y187 yeast was used as a positive control (+), and pGBKT7-Lam / pGADT7-SV40 was used as a negative control (-).

All the LaJAZ proteins contain a conserved TIFY motif in the ZIM domain. Therefore, Y2H experiments were performed to determine whether these proteins interact with each other to form homo- or heterodimers. As shown in Fig 5, some of the LaJAZ proteins showed homo- or heterodimeric interactions, determined as X-α-gal reporter activity. Out of the 7 proteins, three of them exhibited homodimer interactions, in which LaJAZ6 strongly interacted as homodimers, while LaJAZ1 and LaJAZ7 showed weak homomeric interactions. Heterodimeric interactions were observed among LaJAZ1, LaJAZ3, LaJAZ4, LaJAZ5, LaJAZ6 and LaJAZ7, whereas combinations of LaJAZ2 showed no interaction (Fig 5). LaJAZ1 and LaJAZ3 showed the same interaction patterns of heterodimeric interactions, in which they both interacted with LaJAZ4 as the bait, and interacted with LaJAZ4, LaJAZ6 and LaJAZ7 as the prey. LaJAZ4 interacted strongly with LaJAZ1, LaJAZ3 and LaJAZ7 as both the prey and the bait. As a prey, LaJAZ5 interacted with LaJAZ4, LaJAZ6 and LaJAZ7, but no interaction was observed with LaJAZ5 as the bait. In addition, both LaJAZ6 and LaJAZ7 extensively interacted with other members, whereas only a homodimer interaction was observed with LaJAZ6 as the prey.

## Discussion

JAs regulate many aspects of plant growth, development, and defense [1–3]. Acting as transcriptional repressors of JA-responsive genes, the JAZ proteins play a central role in the plant-specific JA signalling pathway [3,4,9,10]. To date, limited information is available about the expression and functions of *JAZ* gene family in *L*. *aurea*. In this study, based on our previous transcriptome data of *L*. *aurea* [39], we identified seven *JAZ* gene members by tblastn search and gene cloning (S1 Table; Fig 1). To further investigate the primary protein structure and evolutionary relationships of LaJAZ proteins, multiple sequence alignment and phylogenetic analyses were performed. The identified LaJAZ proteins contain the conserved TIFY/ZIM domain and Jas motif, and can be divided into three groups (II to IV) (Fig 2). The TIFY/ZIM domain mediates homo- and heteromeric interactions between JAZ proteins [45] and the interaction with NINJA [18,19]. In addition, a subset of JAZ proteins (e.g., *Arabidopsis* JAZ7 and JAZ8) contain N-terminal LxLxL type of EAR motifs that can bind TPL directly to repress JA responses in independence upon NINJA [20,21]. In this regard, LaJAZ4 also displays the LxLxL type of EAR-motif at the N-terminus (S2 Fig) suggesting that LaJAZ4 would be implicated in the TPL-recruited repression machinery without NINJA. The Jas motif consists of a conserved sequence (SLX_2_FX_2_KRX_2_RX_5_PY) at the C-terminal region and is the hallmark feature of the JAZ family that is involved in JA-Ile perception, MYC transcription factor binding, and nuclear localization [3,10,16]. In addition, PPD subfamily proteins often possess three domains: a unique N-terminal PPD domain, a TIFY domain and an abnormal Jas motif lacking the conserved PY amino acids at the C-terminus [11]. Because of the alternative splicing involving intron retention, some splice variants of JAZ proteins (e.g., JAZ10.3) also lack the X_5_PY sequence in the Jas motif, and have a reduced capacity to form complexes with COI1 [46]. We also noticed that LaJAZ6 lack the conserved C-terminal PY residues in the Jas motif, while no PPD domain was found (Fig 1C). Thus, LaJAZ6 may not be regarded as a member of PPD subfamily. However, whether LaJAZ6 is a splice variant should be further investigated.

Our findings indicated that seven *LaJAZ* genes were constitutively expressed in all five *L*. *aurea* tissues (Fig 3A). For example, *LaJAZ1*, *LaJAZ3*, and *LaJAZ4* showed the lowest expression level in *L*. *aurea* leaves, while the expression levels of *LaJAZ2*, *LaJAZ5* and *LaJAZ6* could be highest in the same tissue, suggesting that *LaJAZ2*, *LaJAZ5* and *LaJAZ6* may play an important role in leaf for perception of a jasmonate signal. In addition, each *LaJAZ* gene was differentially expressed in the plant tissues. Such differential expression patterns suggest that the transcription of these seven *LaJAZ* genes would subject to different regulatory mechanisms in *L*. *aurea* tissues. Similarly, previous studies also indicated that *JAZ* genes are differentially and constitutively expressed in different plant species such as rice [15], cotton [47], maize [48], rubber tree [49] and sugarcane [50]. Moreover, *JAZ* genes could be significantly induced by MeJA treatment [17,47,50,51]. In this study, the inducible expression pattern was also demonstrated either *LaJAZ3* in short-term or *LaJAZ1*, *LaJAZ2*, *LaJAZ4* and *LaJAZ7* in long-term plus a delayed *LaJAZ5* response to the MeJA treatment (Fig 3B), suggesting that LaJAZ proteins could be correlated with the jasmonate response under different regulatory mechanisms, in terms of both the jasmonate signaling pathway and the negative feedback, towards the *LaJAZ* expression. Previous studies have shown that JAZ proteins were localized in the nucleus [9,10,52–54]. However, the diverse localization of JAZ proteins was also observed. For example, sugarcane JAZ6 was located in the cytoplasm and the plasma membrane [50]. Rice JAZ1 was found to be localized in cytoplasm, and could function as a nuclear protein in the presence of JA signaling [55]. Our results also showed that LaJAZ3, LaJAZ4, LaJAZ6 and LaJAZ7 was located in nucleus, whereas LaJAZ2 and LaJAZ5 was located in cytoplasm. In addition, LaJAZ1 was localized in both nucleus and cytoplasm (Fig 4). Further studies should be conducted to determine whether the cytoplasm of LaJAZ proteins could be affected by JA signaling. Additionally, yeast two-hybrid assay would demonstrate that homo- or heterodimeric interactions were formed between LaJAZ proteins (Fig 5). We found that LaJAZ4 and LaJAZ7 could interact widely with other LaJAZ proteins, but no interaction took place with LaJAZ2, implying at least that LaJAZ proteins would display isoform selectivity in formation of heterodimers and homodimers. Further studies should be conducted to verify the network of interactions of LaJAZ proteins *in vivo* for figuring out potentials of LaJAZ formation in the jasmonate signaling. Much evidence suggests that JAZ proteins were involved in mediating the biosynthesis of specific secondary metabolites [56–60]. For example, JA enhances the accumulation of anthocyanin [61], and JAZ proteins interact with transcription factors or energy sensor SNF1-RELATED KINASE 1 (SnRK1) to affect anthocyanin accumulation [56,57]. In *Salvia miltiorrhiza* hairy roots, JA-induced tanshinone biosynthesis is mediated by JAZ proteins and SmJAZ8 acts as a core repressor [59,60]. Also, it has been reported that the enhanced accumulation of Amaryllidaceae alkaloids can be elicited by addition of exogenous JAs treatment in Amaryllidaceae plants [37,38,62,63], but the exact molecular regulation mechanisms, including those related with JAZs, are still obscure in this plant family. As a key regulator in plant jasmonate signal pathway, JAZ proteins should play important regulatory role in Amaryllidaceae alkaloids biosynthesis. Thus, the achievement from comprehensive studies of the *JAZ* genes in *L*. *aurea* of Amaryllidacead plants will help us to understand the molecular mechanism of Amaryllidaceae alkaloids biosynthesis.

## Supporting information

S1 Table A list of candidate *LaJAZ* and *LaTIFY* genes in transcriptome data of *L*. *aurea* after challenging with MeJA for 6 h. In our previous transcriptomics study, after correction, the unigenes with a false discovery rate (FDR) ≤ 0.001 and the two-fold change of reads per kb per million reads (RPKM) between two samples were considered as differentially expressed genes (DEGs) [39]. The gene ID of unigenes labeled as blue indicates the PCR-cloned transcript of each *LaJAZ* gene. * represent DEGs.
(PDF)

S2 Table List and information of primers used in this study. (PDF)

S3 Table GenBank accession numbers of JAZ proteins used in this study. Genome databases: *Arabidopsis thaliana* (TAIR, http://www.arabidopsis.org/), *Vitis vinifera* (Grape genome database, http://www.genoscope.cns.fr/externe/GenomeBrowser/Vitis/), *Solanum lycopersicum* (SolGenomics Network, https://www.solgenomics.net/), *Oryza sativa* (Rice genome annotation project, http://rice.plantbiology.msu.edu/), *Zea mays* (MaizeGDB, https://www.maizegdb.org/).
(PDF)

S1 Fig Comparison of LxLxL type EAR motifs of AtJAZ7, AtJAZ8, AtJAZ13 and LaJAZ4. (TIFF)

S2 Fig Assembly strategy for constructs of *LaJAZs* for transient expression in *Arabidopsis* protoplasts. D35S, Double Cauliflower Mosiac Virus 35S; 3’-nos, Nopaline synthase terminator; MCS, multiple cloning site; 5’UTR.
(TIFF)

S3 Fig Immunoblot analysis of LaJAZ1-GFP, LaJAZ2-GFP and LaJAZ5-GFP after protoplast transfection. Red arrow indicates the position of each predicted protein.
(TIFF)

## References

[1] Wasternack C, Hause B. Jasmonates: biosynthesis, perception, signal transduction and action in plant stress response, growth and development. An update to the 2007 review in Annals of Botany. Ann. Bot. 2013; 111(6):1021–1058. 10.1093/aob/mct067 23558912 PMC3662512

[2] Huang H, Liu B, Liu L, Song S. Jasmonate action in plant growth and development. J. Exp. Bot. 2017; 68(6):1349–1359. 10.1093/jxb/erw495 28158849

[3] Howe GA, Major IT, Koo AJ. Modularity in jasmonate signaling for multistress resilience. Annu. Rev. Plant Biol. 2018; 69:387–415. 10.1146/annurev-arplant-042817-040047 29539269

[4] Fonseca S, Chico JM, Solano R. The jasmonate pathway: the ligand, the receptor and the core signalling module. Curr. Opin. Plant Biol. 2009; 12(5):539–547. 10.1016/j.pbi.2009.07.013 19716757

[5] Chini A, Monte I, Zamarreño AM, Hamberg M, Lassueur S, Reymond P, et al An OPR3-independent pathway uses 4,5-didehydrojasmonate for jasmonate synthesis. Nat. Chem. Biol. 2018; 14:171–178. 10.1038/nchembio.2540 29291349

[6] Wasternack C, Hause B. A bypass in jasmonate biosynthesis—the OPR3-independent formation. Trends Plant Sci. 2018; 23(4):276–279. 10.1016/j.tplants.2018.02.011 29530379

[7] Howe GA, Yoshida Y. Evolutionary origin of JAZ proteins and jasmonate signaling. Mol. Plant. 2019; 12(2):153–155. 10.1016/j.molp.2019.01.015 30690172

[8] Monte I, Ishida S, Zamarreño AM, Hamberg M, Franco-Zorrilla JM, García-Casado G, et al Ligand-receptor co-evolution shaped the jasmonate pathway in land plants. Nat. Chem. Biol. 2018; 14:480–488. 10.1038/s41589-018-0033-4 29632411

[9] Chini A, Fonseca S, Fernández G, Adie B, Chico JM, Lorenzo O, et al The JAZ family of repressors is the missing link in jasmonate signaling. Nature. 2007; 448(7154):666–671. 10.1038/nature06006 17637675

[10] Thines B, Katsir L, Melotto M, Niu Y, Mandaokar A, Liu G, et al JAZ repressor proteins are targets of the SCF^COI1^ complex during jasmonate signaling. Nature. 2007; 448 (7154):661–665. 10.1038/nature05960 17637677

[11] Bai Y, Meng Y, Huang D, Qi Y, Chen M. Origin and evolutionary analysis of the plant-specific TIFY transcription factor family. Genomics. 2011; 98(2):128–136. 10.1016/j.ygeno.2011.05.002 21616136

[12] Monte I, Franco-Zorrilla JM, García-Casado G, Zamarreño AM, García-Mina JM, Nishihama R, et al A single JAZ repressor controls the jasmonate pathway in *Marchantia polymorpha*. Mol. Plant. 2019; 12(2):185–198. 10.1016/j.molp.2018.12.017 30594656

[13] Chini A, Gimenez-Ibanez S, Goossens A, Solano R. Redundancy and specificity in jasmonate signaling. Curr. Opin. Plant Biol. 2016; 33:147–156. 10.1016/j.pbi.2016.07.005 27490895

[14] Thireault C, Shyu C, Yoshida Y, St Aubin B, Campos ML, Howe GA. Repression of jasmonate signaling by a non-TIFY JAZ protein in Arabidopsis. Plant J. 2015; 82(4):669–679. 10.1111/tpj.12841 25846245

[15] Ye H, Du H, Tang N, Li X, Xiong L. Identification and expression profiling analysis of *TIFY* family genes involved in stress and phytohormone responses in rice. Plant Mol. Biol. 2009; 71(3):291–305. 10.1007/s11103-009-9524-8 19618278

[16] Melotto M, Mecey C, Niu Y, Chung HS, Katsir L, Zeng W, et al A critical role of two positively charged amino acids in the Jas motif of Arabidopsis JAZ proteins in mediating coronatine- and jasmonoyl isoleucine-dependent interactions with the COI1 F-box protein. Plant J. 2008; 55(6):979–988. 10.1111/j.1365-313X.2008.03566.x 18547396 PMC2653208

[17] Yan Y, Stolz S, Chételat A, Reymond P, Pagni M, Dubugnon L, et al, A downstream mediator in the growth repression limb of the jasmonate pathway. Plant Cell. 2007; 19(8):2470–2483. 10.1105/tpc.107.050708 17675405 PMC2002611

[18] Pauwels L, Barbero GF, Geerinck J, Tilleman S, Grunewald W, Pérez AC, et al NINJA connects the co-repressor TOPLESS to jasmonate signaling. Nature. 2010; 464(7289):788–791. 10.1038/nature08854 20360743 PMC2849182

[19] Pauwels L, Goossens A. The JAZ Proteins: a crucial interface in the jasmonate signaling cascade. Plant Cell. 2011; 23(9):3089–3100. 10.1105/tpc.111.089300 21963667 PMC3203442

[20] Shyu C, Figueroa P, Depew CL, Cooke TF, Sheard LB, Moreno JE, et al JAZ8 lacks a canonical degron and has an EAR motif that mediates transcriptional repression of jasmonate responses in Arabidopsis. Plant Cell. 2012; 24(2):536–550. 10.1105/tpc.111.093005 22327740 PMC3315231

[21] Thatcher LF, Cevik V, Grant M, Zhai B, Jones JD, Manners JM, et al Characterization of a JAZ7 activation-tagged *Arabidopsis* mutant with increased susceptibility to the fungal pathogen *Fusarium oxysporum*. J. Exp. Bot. 2016; 67(8):2367–2386. 10.1093/jxb/erw040 26896849 PMC4809290

[22] He M, Qu C, Gao O, Hu X, Hong X. Biological and pharmacological activities of Amaryllidaceae alkaloids. RSC Adv. 2015; 5(21):16562–16574.

[23] Kurita S. Variation and evolution on the karyotype of *Lycoris*, Amaryllidaceae II. Karyotype analysis of ten taxa among which seven are native in China. Cytologia. 1987; 52:19–40.

[24] Huang XA, Dong MF, Wang XH, Shang FD. Chromosome report of *Lycoris* Herb. (Amaryllidaceae). J. Syst. Evol. 2011; 49(2):164.

[25] Chang YC, Shii CT, Lee YC, Chung MC. Diverse chromosome complements in the functional gametes of interspecific hybrids of MT- and A-karyotype *Lycoris* spp. Plant Syst. Evol. 2013; 299:1141–1155.

[26] Ru Q, Wang X, Liu T, Zheng H. Physiological and comparative proteomic analyses in response to nitrogen application in an Amaryllidaceae plant, *Lycoris aurea*. Acta Physiol. Plant. 2013; 35:271–282.

[27] Quan M, Liang J. The influences of four types of soil on the growth, physiological and biochemical characteristics of *Lycoris aurea* (L’ Her.) Herb. Sci, Rep. 2017; 7:43284. 28240308 10.1038/srep43284 PMC5327428

[28] Yang Y, Huang SX, Zhao YM, Zhao QS, Sun HD. Alkaloids from the bulbs of *Lycoris aurea*. Helv. Chim. Acta. 2005; 88:2550–2553.

[29] Pi HF, Zhang P, Ruan HL, Zhang YH, Sun HD, Wu JZ. A new alkaloid from *Lycoris aurea*. Chinese Chem. Lett. 2009; 20(11):1319–1320.

[30] Tian Y, Zhang C, Guo M. Comparative analysis of Amaryllidaceae alkaloids from three *Lycoris* species. Molecules. 2015; 20:21854–21869. 10.3390/molecules201219806 26690108 PMC6332018

[31] Liao N, Ao M, Zhang P, Yu L. Extracts of *Lycoris aurea* induce apoptosis in *Murine Sarcoma* S180 cells. Molecules. 2012; 17:3723–3735. 10.3390/molecules17043723 22450682 PMC6268187

[32] Song JH, Zhang L, Song Y. Alkaloids from *Lycoris aurea* and their cytotoxicities against the head and neck squamous cell carcinoma. Fitoterapia. 2014; 95:121–126. 10.1016/j.fitote.2014.03.006 24631767

[33] Liu J, Xu X, Liu J, Balzarini J, Luo Y, Kong Y, et al A novel tetrameric lectin from *Lycoris aurea* with four mannose binding sites per monomer. Acta Biochim. Pol. 2007; 54 (1):159–166. 17356714

[34] Li Y, Li J, Qian B, Cheng L, Xu S, Wang R. De Novo biosynthesis of *p*-coumaric acid in *E*. *coli* with a *trans*-cinnamic acid 4-hydroxylase from the Amaryllidaceae plant *Lycoris aurea*. Molecules. 2018; 23(12):3185. 10.3390/molecules23123185 PMC6320932 30513965

[35] Sun B, Wang P, Wang R, Li Y, Xu S. Molecular cloning and characterization of a *meta*/*para*-*O*-methyltransferase from *Lycoris aurea*. Int. J. Mol. Sci. 2018; 19(7):1911. 10.3390/ijms19071911 PMC6073595 29966257

[36] Wang R, Han X, Xu S, Xia B, Jiang Y, Xue Y, et al Cloning and characterization of a tyrosine decarboxylase involved in the biosynthesis of galanthamine in *Lycoris aurea*. PeerJ. 2019; 7:e6729 10.7717/peerj.6729 31024762 PMC6474336

[37] Mu HM, Wang R, Li XD, Jiang YM, Wang CY, Quan JP, et al Effect of abiotic and biotic elicitors on growth and alkaloid accumulation of *Lycoris chinensis* seedlings. Z. Naturforsch. C. 2009; 64(7–8):541–550. 10.1515/znc-2009-7-813 19791507

[38] Jiang Y, Xia N, Li X, Shen W, Liang L, Wang C, et al Molecular cloning and characterization of a phenylalanine ammonia-lyase gene (*LrPAL*) from *Lycoris radiata*. Mol. Biol. Rep. 2011; 38(3):1935–1940. 10.1007/s11033-010-0314-9 20857216

[39] Wang R, Xu S, Wang N, Xia B, Jiang Y, Wang Ren. Transcriptome analysis of secondary metabolism pathway, transcription factors, and transporters in response to methyl jasmonate in *Lycoris aurea*. Front. Plant Sci. 2017; 7:1971 10.3389/fpls.2016.01971 28111578 PMC5217099

[40] Xu S, Jiang Y, Wang N, Xia B, Jiang Y, Li X, et al Identification and differential regulation of microRNAs in response to methyl jsamonate treatment in *Lycoris aurea* by deep sequencing. BMC Genomics. 2016; 17:789 10.1186/s12864-016-2645-y 27724902 PMC5057397

[41] Sievers F, Wilm A, Dineen D, Gibson TJ, Karplus K, Li W, et al Fast, scalable generation of high-quality protein multiple sequence alignments using Clustal Omega. Mol. Syst. Biol. 2011; 7:539 10.1038/msb.2011.75 21988835 PMC3261699

[42] Wu FH, Shen SC, Lee LY, Lee SH, Chan MT, Li CS. Tape-*Arabidiopsis* Sandwich—a simpler *Arabidopsis* protoplast isolation method. Plant Methods. 2009; 5:16 10.1186/1746-4811-5-16 19930690 PMC2794253

[43] Ma R, Xu S, Zhao Y, Xia B, Wang R. Selection and validation of appropriate reference genes for quantitative real-time PCR analysis of gene expression in *Lycoris aurea*. Front. Plant Sci. 2016; 7:536 10.3389/fpls.2016.00536 27200013 PMC4843812

[44] Pedersen DS, Merkle T, Marktl B, et al Nucleocytoplasmic distribution of the Arabidopsis chromatin-associated HMGB2/3 and HMGB4 proteins. Plant Physiol. 2010; 154:1831–1841. 10.1104/pp.110.163055 20940346 PMC2996034

[45] Chini A, Fonseca S, Chico JM, Fernández-Calvo P, Solano R. The ZIM domain mediates homo- and heteromeric interactions between Arabidopsis JAZ proteins. Plant J. 2009; 59(1):77–87. 10.1111/j.1365-313X.2009.03852.x 19309455

[46] Chung HS, Cooke TF, DePew CL, Patel LC, Ogawa N, Kobayashi Y, et al Alternative splicing expands the repertoire of dominant JAZ repressors of jasmonate signaling. Plant J. 2010; 63:613–622. 10.1111/j.1365-313X.2010.04265.x 20525008 PMC2966510

[47] Sun Q, Wang G, Zhang X, Zhang X, Qiao P. Long L, et al Genome-wide identification of the TIFY gene family in three cultivated *Gossypium* species and the expression of JAZ genes. Sci. Rep. 2017; 7:42418 10.1038/srep42418 28186193 PMC5301204

[48] Zhou X, Yan S, Sun C, Li S, Li J, Xu M, et al A maize jasmonate Zim-domain protein, ZmJAZ14, associates with the JA, ABA, and GA signaling pathways in transgenic *Arabidopsis*. PLoS One. 2015; 10(3):e0121824 10.1371/journal.pone.0121824 25807368 PMC4373942

[49] Hong H, Xiao H, Yuan H, Zhai J, Huang X. Cloning and characterisation of *JAZ* gene family in *Hevea Brasiliensis*. Plant Biol. 2015; 17(3):618–624. 10.1111/plb.12288 25399518

[50] Liu F, Sun T, Wang L, Su W, Gao S, Su Y, et al Plant jasmonate ZIM domain genes: shedding light on structure and expression patterns of *JAZ* gene family in sugarcane. BMC Genomics. 2017; 18(1):771 10.1186/s12864-017-4142-3 29020924 PMC5637078

[51] Sun H, Chen L, Li JY, Hu ML, Ullah A, He X, et al The JASMONATE ZIM-domain gene family mediates JA signaling and stress response in cotton. Plant Cell Physiol. 2017; 58(12):2139–2154. 10.1093/pcp/pcx148 29036515

[52] Chung HS, Howe GA. A critical role for the TIFY motif in repression of jasmonate signaling by a stabilized splice variant of the JASMONATE ZIM-domain protein JAZ10 in *Arabidopsis*. Plant Cell. 2009; 21(1):131–145. 10.1105/tpc.108.064097 19151223 PMC2648087

[53] Grunewald W, Vanholme B, Pauwels L, Plovie E, Inzé D, Gheysen G, et al Expression of the Arabidopsis jasmonate signaling repressor JAZ1/TIFY10A is stimulated by auxin. EMBO Rep. 2009; 10(8):923–928. 10.1038/embor.2009.103 19575013 PMC2726675

[54] Withers J, Yao J, Mecey C, Howe GA, Melotto M, He SY. Transcription factor-dependent nuclear localization of a transcriptional repressor in jasmonate hormone signaling. Proc. Natl. Acad. Sci. U.S.A. 2012; 109(49):20148–20153. 10.1073/pnas.1210054109 23169619 PMC3523844

[55] Fu J, Wu H, Ma S, Xiang D, Liu R, Xiong L. OsJAZ1 attenuates drought resistance by regulating JA and ABA signaling in rice. Front. Plant Sci. 2017; 8:2108 10.3389/fpls.2017.02108 29312378 PMC5733117

[56] Qi T, Song S, Ren Q, Wu D, Huang H, Chen Y, et al The jasmonate-ZIM-domain proteins interact with the WD-repeat/bHLH/MYB complexes to regulate jasmonate-mediated anthocyanin accumulation and trichome initiation in *Arabidopsis thaliana*. Plant Cell. 2011; 23(5):1795–1814. 10.1105/tpc.111.083261 21551388 PMC3123955

[57] Liu XJ, An XH, Liu X, Hu DG, Wang XF, You CX, et al MdSnRK1.1 interacts with MdJAZ18 to regulate sucrose-induced anthocyanin and proanthocyanidin accumulation in apple. J. Exp. Bot. 2017; 68(11):2977–2990. 10.1093/jxb/erx150 28549152 PMC5853841

[58] Zhang HB, Bokowiec MT, Rushton PJ, Han SC, Timko MP. Tobacco transcription factors NtMYC2a and NtMYC2b form nuclear complexes with the NtJAZ1 repressor and regulate multiple jasmonate-inducible steps in nicotine biosynthesis. Mol. Plant. 2012; 5(1):73–84. 10.1093/mp/ssr056 21746701

[59] Shi M, Zhou W, Zhang J, Huang S, Wang H, Kai G, et al Methyl jasmonate induction of tanshinone biosynthesis in *Salvia miltiorrhiza* hairy roots is mediated by JASMONATE ZIM-DOMAIN repressor proteins. Sci. Rep. 2016; 6:20919 10.1038/srep20919 26875847 PMC4753458

[60] Pei T, Ma P, Ding K, Liu S, Jia Y, Ru M, et al SmJAZ8 acts as a core repressor regulating JA-induced biosynthesis of salvianolic acids and tanshinones in *Salvia miltiorrhiza* hairy roots. J. Exp. Bot. 2018; 69(7):1663–1678. 10.1093/jxb/erx484 29281115

[61] Li T, Jia KP, Lian HL, Yang X, Li L, Yang HQ. Jasmonic acid enhancement of anthocyanin accumulation is dependent on phytochrome A signaling pathway under far-red light in *Arabidopsis*. Biochem. Biophy. Res. Commun. 2014; 454(1):78–83. 10.1016/j.bbrc.2014.10.059 25450360

[62] Colque R, Viladomat F, Bastida J, Codina C. Improved production of galanthamine and related alkaloids by methyl jasmonate in *Narcissus* confuses shoot-clumps. Planta Med. 2004; 70(12):1180–1188. 10.1055/s-2004-835849 15643555

[63] Ivanov I, Georgiev V, Pavlov A. Elicitation of galanthamine biosynthesis by *Leucojum aestivum* liquid shoot cultures. J. Plant Physiol. 2013; 170(12):1122–1129. 10.1016/j.jplph.2013.03.017 23648110

