# Supplementary material for: Identification, molecular characterization and expression of JAZ genes in Lycoris aurea
Source: PLoS One. 2020 Mar 17;15(3):e0230177. doi: 10.1371/journal.pone.0230177 (PMC7077819; doi:10.1371/journal.pone.0230177)
Supplement: S1 Table — In our previous transcriptomics study, after correction, the unigenes with a false discovery rate (FDR) ≤ 0.001 and the two-fold change of reads per kb per million reads (RPKM) between two samples were considered as differentially expressed genes (DEGs) [39]. The gene ID of unigenes labeled as blue indicates the PCR-cloned transcript of each LaJAZ gene. * represent DEGs. (PDF) [file pone.0230177.s001.pdf]

S1 Table. A list of candidate *LaJAZ* genes in *L. aurea* after treating with MeJA for 6 h.

Gene name	Gene ID	Gene length	Control_ RPKM	MeJA_ RPKM	Log_2 fold change (MeJA/Control)	Regulation
LaJAZ1	CL8438.Contig3_All	994	8.0445	18.8225	1.226384	Up-regulation*
	Unigene17506_All	1044	11.1406	10.3942	-0.10005	
	Unigene17507_All	1044	0.116	2.1505	4.212475	
	CL8438.Contig1_All	995	61.8557	190.5427	1.623136	
LaJAZ2	CL4779.Contig5_All	879	4.135	2.1285	-0.95805	Down-regulation
	CL4779.Contig1_All	1090	2.5565	1.2587	-1.02224	
	CL4779.Contig3_All	794	1.5259	0.4713	-1.69494	
LaJAZ3	CL2513.Contig1_All	834	62.0299	390.642	2.654811	Up-regulation*
	CL2513.Contig2_All	1038	60.3437	181.5678	1.589233	
	CL2513.Contig3_All	789	2.3033	12.0146	2.383014	
	CL2513.Contig4_All	974	10.9462	93.2275	3.090325	
LaJAZ4	CL5980.Contig3_All	545	2.223	51.0364	4.520947	Up-regulation*
	CL5980.Contig2_All	665	38.4415	380.568	3.307418	
LaJAZ5	CL6538.Contig1_All	1690	142.0875	191.5975	0.431299	Up-regulation
	CL6538.Contig2_All	573	46.9394	31.9989	-0.55278	
LaJAZ6	Unigene8793_All	1558	11.8199	9.0465	-0.38579	Down-regulation
LaJAZ7	CL4538.Contig4_All	1124	204.4749	470.8456	1.20333	Up-regulation*
	CL4538.Contig2_All	1140	55.3697	88.2959	0.67325	
	CL4538.Contig3_All	1115	35.64	98.1062	1.460847	
LaTIFY1	CL6481.Contig2_All	1024	29.5787	51.2807	0.793857	Up-regulation
	CL6481.Contig1_All	654	15.0054	17.1647	0.193963	
LaTIFY2	CL9072.Contig1_All	1267	17.0209	15.5544	-0.12998	Down-regulation
