# Supplementary material for: Identification, molecular characterization and expression of JAZ genes in Lycoris aurea
Source: PLoS One. 2020 Mar 17;15(3):e0230177. doi: 10.1371/journal.pone.0230177 (PMC7077819; doi:10.1371/journal.pone.0230177)
Supplement: S2 Table — (PDF) [file pone.0230177.s002.pdf]

S2 Table. List and information of primers used in this study.

Primer names	Forward primer (5'–3')	Reverse primer (5'–3')	Usage
LaJAZ1-AD	GTACCAGATTACGCTCATATGTCTGAGAAGAAGTCTACCTT	CGATTCATCTGCAGCTGCAGCTATGAGCTCGAGCTGGAAT	Y2H assay
LaJAZ2-AD	GTACCAGATTACGCTCATATGGATCTCTCAGAATCCCAAAT	CGATTCATCTGCAGCTGCAGTTAGATCTGAGGCTTCACTT	Y2H assay
LaJAZ3-AD	GTACCAGATTACGCTCATATGGCTGAGATCAAGAAATC	CGATTCATCTGCAGCTGCAGTTAGTTCACATTTGAGATCT	Y2H assay
LaJAZ4-AD	GTACCAGATTACGCTCATATGGAGAATGAGTATGATGACGA	CGATTCATCTGCAGCTGCAGCTAAGTTGGTCTACACCCA	Y2H assay
LaJAZ5-AD	GTACCAGATTACGCTCATATGGAGAGGGACTTCATGGGAAT	CGATTCATCTGCAGCTGCAGTTACATCTCTAATTTAGTAC	Y2H assay
LaJAZ6-AD	GTACCAGATTACGCTCATATGAGTAGTAGTGGCAGCA	CGATTCATCTGCAGCTCGAGTTATTCGCAACCGCCATAATCG	Y2H assay
LaJAZ7-AD	GTACCAGATTACGCTCATATGTCGAAGATTACGAAGCAAG	CGATTCATCTGCAGCTGCAGTTATCTTCTGCAATCTGAGCTCA	Y2H assay
LaJAZ1-BD	TCAGAGGAGGACCTGCATATGTCTGAGAAGAAGTCTACCTT	CTAGTTATGCGGCCGCTGCAGCTATGAGCTCGAGCTGGAAT	Y2H assay
LaJAZ2-BD	TCAGAGGAGGACCTGCATATGGATCTCTCAGAATCCCAAAT	CTAGTTATGCGGCCGCTGCAGTTAGATCTGAGGCTTCACTT	Y2H assay
LaJAZ3-BD	TCAGAGGAGGACCTGCATATGGCTGAGATCAAGAAATC	CTAGTTATGCGGCCGCTGCAGTTAGTTCACATTTGAGATCT	Y2H assay
LaJAZ4-BD	TCAGAGGAGGACCTGCATATGGAGAATGAGTATGATGACGA	CTAGTTATGCGGCCGCTGCAGCTAAGTTGGTCTACACCCA	Y2H assay
LaJAZ5-BD	TCAGAGGAGGACCTGCATATGGAGAGGGACTTCATGGGAAT	CTAGTTATGCGGCCGCTGCAGTTACATCTCTAATTTAGTAC	Y2H assay
LaJAZ6-BD	TCAGAGGAGGACCTGCATATGAGTAGTAGTGGCAGCA	CTAGTTATGCGGCCGCTGCAGTTATTCGCAACCGCCATAATCG	Y2H assay
LaJAZ7-BD	TCAGAGGAGGACCTGCATATGTCGAAGATTACGAAGCAAG	CTAGTTATGCGGCCGCTGCAGTTATCTTCTGCAATCTGAGCTCA	Y2H assay
LaJAZ1-GFP	AGGACCGGTCCCGGGGGATCCATGTCTGAGAAGAAGTCTAC	GCCCTTGCTCACCATGGATCCTGAGCTCGAGCTGGAAT	Subcellular localization assay
LaJAZ2-GFP	AGGACCGGTCCCGGGGGATCCATGGATCTCTCAGAATCCCAAA	GCCCTTGCTCACCATGGATCCGATCTGAGGCTTCACTT	Subcellular localization assay
LaJAZ3-GFP	AGGACCGGTCCCGGGGGATCCATGGCTGAGATCAAGAAATC	GCCCTTGCTCACCATGGATCCGTTCACATTTGAGATCT	Subcellular localization assay
LaJAZ4-GFP	AGGACCGGTCCCGGGGGATCCATGGAGAATGAGTATGATGACGA	GCCCTTGCTCACCATGGATCCAGTTGGTCTACACCCATAA	Subcellular localization assay
LaJAZ5-GFP	AGGACCGGTCCCGGGGGATCCATGGAGAGGGACTT	GCCCTTGCTCACCATGGATCCCATCTCTAATTTAGTACTTG	Subcellular localization assay
LaJAZ6-GFP	AGGACCGGTCCCGGGGGATCCATGAGTAGTAGTGGCAGCA	GCCCTTGCTCACCATGGATCCTTCGCAACCGCCATAATCG	Subcellular localization assay
LaJAZ7-GFP	AGGACCGGTCCCGGGGGATCCATGTCGAAGATTACGAAGCAAG	GCCCTTGCTCACCATGGATCCTCTTCTGCAATCTGAGCTCA	Subcellular localization assay
AtHMGB1-mCherry	CGCTGGATCCATGAAAACAGCAAAGGGGAAAGAT	AATCCCCGGGGTCTTCTTCCTCGTCGTCAT	Nucleus-localized marker construction
LaJAZ1-qPCR	AGAACAAACCAAAGGTTTATTTAGG	TTATCGAAGACCAACACTTTGCCAT	qRT-PCR
LaJAZ2-qPCR	GTTCTTCTCCGCCGCTGCCGTCTGGT	CTGAAGCATTGGGACCTGCAGTGATTG	qRT-PCR
LaJAZ3-qPCR	AAATCAATACTTCTGGTATCCCTAT	TTGCATCTTCCCTTCTGAAATTAGT	qRT-PCR
LaJAZ4-qPCR	GAGCTCCAGCTCGGCAGCGGCCAT	GCCATACGTATTATAGCTCTTGCCT	qRT-PCR
LaJAZ5-qPCR	CCAAGAGCTATACCTCAGGCTAGA	ACGACTCCTCCCGATTGCTTGAAAGA	qRT-PCR
LaJAZ6-qPCR	CCCAACCCGATTGCCACGCACGCGA	AAGAACTGGTCCAGCAGGCGGGTC	qRT-PCR
LaJAZ7-qPCR	GCTCCACGACCAATCAATTCTTCG	TTTCTGGCTCCCTGCTTTCCGCAAC	qRT-PCR
LaTIP41	GCAACCATCCAAAGTTTAACTGCT	AATGTGCAAGCAGGGCTAGTAA	qRT-PCR
