# Supplementary material for: Identification, molecular characterization and expression of JAZ genes in Lycoris aurea
Source: PLoS One. 2020 Mar 17;15(3):e0230177. doi: 10.1371/journal.pone.0230177 (PMC7077819; doi:10.1371/journal.pone.0230177)
Supplement: S3 Table — Genome databases: Arabidopsis thaliana (TAIR, http://www.arabidopsis.org/), Vitis vinifera (Grape genome database, http://www.genoscope.cns.fr/externe/GenomeBrowser/Vitis/), Solanum lycopersicum (SolGenomics Network, https://www.solgenomics.net/), Oryza sativa (Rice genome annotation project, http://rice.plantbiology.msu.edu/), Zea mays (MaizeGDB, https://www.maizegdb.org/). (PDF) [file pone.0230177.s003.pdf]

S3 Table. GenBank accession numbers of JAZ proteins used in this study.

Protein names	Species	Accession number	Database [a]
AtJAZ1	*Arabidopsis thaliana*	AT1G19180	TAIR
AtJAZ2	*Arabidopsis thaliana*	AT1G74950	TAIR
AtJAZ3	*Arabidopsis thaliana*	AT3G17860	TAIR
AtJAZ4	*Arabidopsis thaliana*	AT1G48500	TAIR
AtJAZ5	*Arabidopsis thaliana*	AT1G17380	TAIR
AtJAZ6	*Arabidopsis thaliana*	AT1G72450	TAIR
AtJAZ7	*Arabidopsis thaliana*	AT2G34600	TAIR
AtJAZ8	*Arabidopsis thaliana*	AT1G30135	TAIR
AtJAZ9	*Arabidopsis thaliana*	AT1G70700	TAIR
AtJAZ10	*Arabidopsis thaliana*	AT5G13220	TAIR
AtJAZ11	*Arabidopsis thaliana*	AT3G43440	TAIR
AtJAZ12	*Arabidopsis thaliana*	AT5G20900	TAIR
AtJAZ13	*Arabidopsis thaliana*	AT3G22275	TAIR
OsJAZ1	*Oryza sative*	LOC_Os04g55920	Rice Genome Anottation Project
OsJAZ2	*Oryza sative*	LOC_Os07g05830	Rice Genome Anottation Project
OsJAZ3	*Oryza sative*	LOC_Os08g33160	Rice Genome Anottation Project
OsJAZ4	*Oryza sative*	LOC_Os09g23660	Rice Genome Anottation Project
OsJAZ5	*Oryza sative*	LOC_Os04g32480	Rice Genome Anottation Project
OsJAZ6	*Oryza sative*	LOC_Os03g28940	Rice Genome Anottation Project
OsJAZ7	*Oryza sative*	LOC_Os07g42370	Rice Genome Anottation Project
OsJAZ8	*Oryza sative*	LOC_Os09g26780	Rice Genome Anottation Project
OsJAZ9	*Oryza sative*	LOC_Os03g08310	Rice Genome Anottation Project
OsJAZ10	*Oryza sative*	LOC_Os03g08330	Rice Genome Anottation Project
OsJAZ11	*Oryza sative*	LOC_Os03g08320	Rice Genome Anottation Project
OsJAZ12	*Oryza sative*	LOC_Os10g25290	Rice Genome Anottation Project
OsJAZ13	*Oryza sative*	LOC_Os10g25230	Rice Genome Anottation Project
OsJAZ14	*Oryza sative*	LOC_Os10g25250	Rice Genome Anottation Project
OsJAZ15	*Oryza sative*	LOC_Os03g27900	Rice Genome Anottation Project
ZmJAZ1	*Zea mays*	GRMZM2G343157_P01	MaizeGDB
ZmJAZ2	*Zea mays*	GRMZM2G445634_P01	MaizeGDB
ZmJAZ3	*Zea mays*	GRMZM2G117513_P01	MaizeGDB
ZmJAZ4	*Zea mays*	GRMZM2G024680_P01	MaizeGDB
ZmJAZ5	*Zea mays*	GRMZM2G145412_P01	MaizeGDB
ZmJAZ6	*Zea mays*	GRMZM2G145458_P01	MaizeGDB
ZmJAZ7	*Zea mays*	GRMZM2G382794_P01	MaizeGDB
ZmJAZ8	*Zea mays*	GRMZM2G086920_P01	MaizeGDB
ZmJAZ9	*Zea mays*	GRMZM2G145407_P01	MaizeGDB
ZmJAZ10	*Zea mays*	GRMZM2G171830_P01	MaizeGDB
ZmJAZ11	*Zea mays*	GRMZM2G005954_P01	MaizeGDB
ZmJAZ12	*Zea mays*	GRMZM2G101769_P01	MaizeGDB
ZmJAZ13	*Zea mays*	GRMZM2G151519_P01	MaizeGDB
ZmJAZ14	*Zea mays*	GRMZM2G064775_P01	MaizeGDB
ZmJAZ15	*Zea mays*	GRMZM2G173596_P01	MaizeGDB
ZmJAZ16	*Zea mays*	GRMZM2G338829_P01	MaizeGDB
ZmJAZ17	*Zea mays*	GRMZM2G126507_P01	MaizeGDB
ZmJAZ18	*Zea mays*	GRMZM2G116614_P01	MaizeGDB
ZmJAZ19	*Zea mays*	GRMZM2G066020_P01	MaizeGDB
ZmJAZ20	*Zea mays*	GRMZM2G089736_P01	MaizeGDB
ZmJAZ21	*Zea mays*	GRMZM2G036351_P01	MaizeGDB
ZmJAZ22	*Zea mays*	GRMZM2G036288_P01	MaizeGDB
ZmJAZ23	*Zea mays*	GRMZM2G143402_P01	MaizeGDB
SlJAZ1	*Solanum lycopersicum*	Solyc07g042170	Sol Genomics Network
SlJAZ2	*Solanum lycopersicum*	Solyc12g009220	Sol Genomics Network

SlJAZ3	*Solanum lycopersicum*	Solyc03g122190	Sol Genomics Network
SlJAZ4	*Solanum lycopersicum*	Solyc12g049400	Sol Genomics Network
SlJAZ5	*Solanum lycopersicum*	Solyc03g118540	Sol Genomics Network
SlJAZ6	*Solanum lycopersicum*	Solyc01g005440	Sol Genomics Network
SlJAZ7	*Solanum lycopersicum*	Solyc11g011030	Sol Genomics Network
SlJAZ8	*Solanum lycopersicum*	Solyc06g068930	Sol Genomics Network
SlJAZ9	*Solanum lycopersicum*	Solyc08g036640	Sol Genomics Network
SlJAZ10	*Solanum lycopersicum*	Solyc08g036620	Sol Genomics Network
SlJAZ11	*Solanum lycopersicum*	Solyc08g036660	Sol Genomics Network
SlJAZ12	*Solanum lycopersicum*	Solyc01g009740	Sol Genomics Network
SlJAZ13	*Solanum lycopersicum*	Solyc01g103600	Sol Genomics Network
VvJAZ1	*Vitis vinifera*	GSVIVG01011679001	Vitis Genoscope
VvJAZ2	*Vitis vinifera*	GSVIVG01000967001	Vitis Genoscope
VvJAZ3	*Vitis vinifera*	GSVIVG01007188001	Vitis Genoscope
VvJAZ4	*Vitis vinifera*	GSVIVG01016721001	Vitis Genoscope
VvJAZ5	*Vitis vinifera*	GSVIVG01021514001	Vitis Genoscope
VvJAZ6	*Vitis vinifera*	GSVIVG01021516001	Vitis Genoscope
VvJAZ7	*Vitis vinifera*	GSVIVG01021518001	Vitis Genoscope
VvJAZ8	*Vitis vinifera*	GSVIVG01021519001	Vitis Genoscope
VvJAZ9	*Vitis vinifera*	GSVIVG01015042001	Vitis Genoscope
VvJAZ10	*Vitis vinifera*	GSVIVG01023256001	Vitis Genoscope
VvJAZ11	*Vitis vinifera*	GSVIVG01008453001	Vitis Genoscope
