# Supplementary figures and images for: Identification, molecular characterization and expression of JAZ genes in Lycoris aurea
Source: PLoS One. 2020 Mar 17;15(3):e0230177. doi: 10.1371/journal.pone.0230177 (PMC7077819; doi:10.1371/journal.pone.0230177)

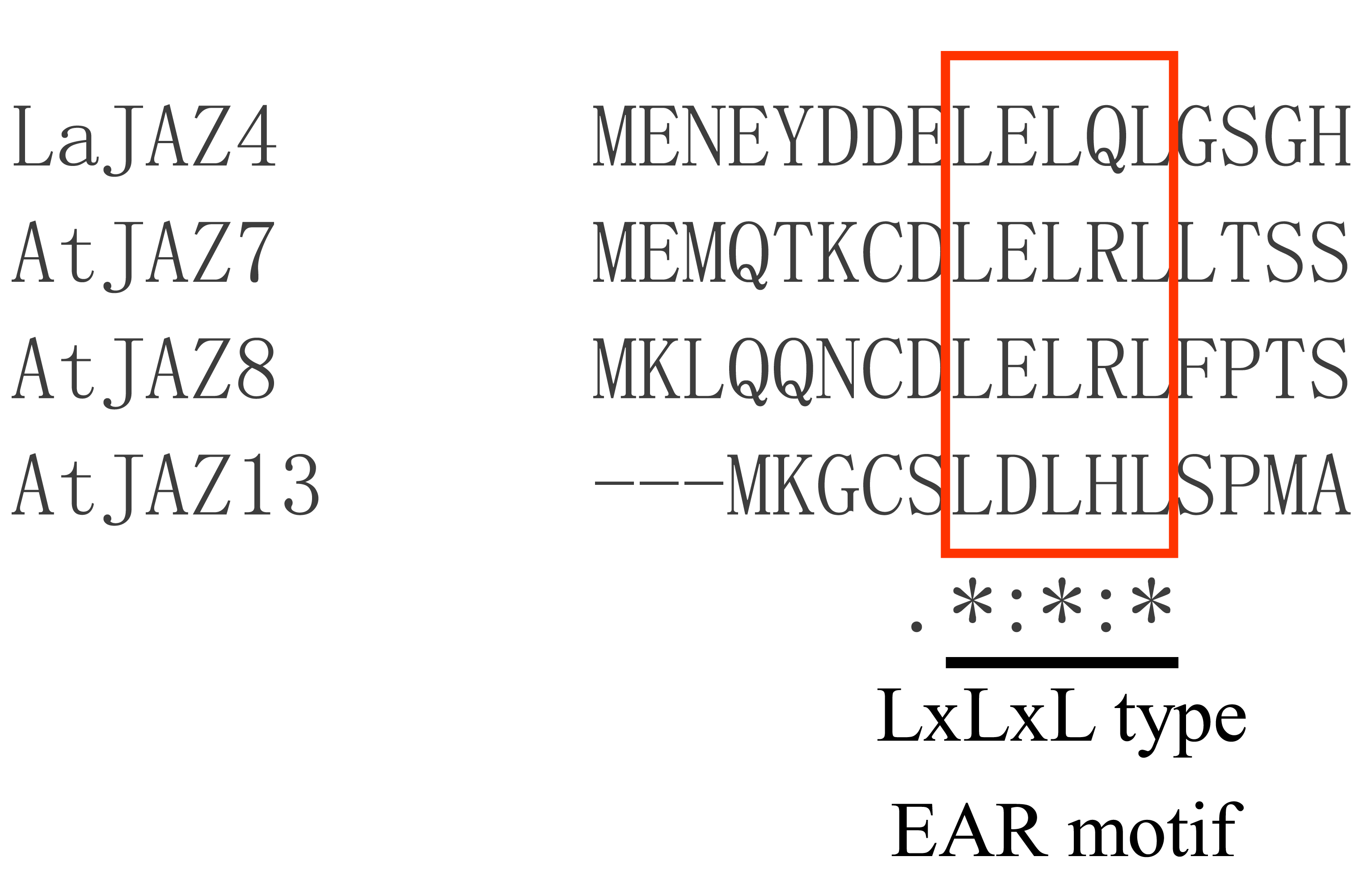

Supplement: S1 Fig — (TIFF) [file pone.0230177.s004.tiff]
